# A coumarin analogue NFA from endophytic *Aspergillus fumigatus* improves drought resistance in rice as an antioxidant

**DOI:** 10.1186/s12866-019-1419-5

**Published:** 2019-02-26

**Authors:** Wanggege Qin, Chengxiong Liu, Wei Jiang, Yanhong Xue, Guangxi Wang, Shiping Liu

**Affiliations:** 1 College of Life Science and Pharmacy, China Three Gorges University, Yichang, 443002 China; 2 Hubei Key Laboratory of Natural Products Research and Development, College of Life Science and Pharmacy, China Three Gorges University, Yichang, 443002 China; 3 Laboratory of Plant Conservation Science, Faculty of Agriculture, Meijo University, Aichi, 468-8502 Japan

**Keywords:** Endophytic fungus, *Aspergillus fumigatus*, NFA, Antioxidant, Drought stress

## Abstract

**Background:**

Drought and its resulting oxidative damage are the major yield limiting factors for crops in arid and semi-arid regions. Recent studies have found that endophytic fungi coexisting in plants can alleviate biotic or abiotic damage to plant growth and development. In order to screen for the endophytes associated with drought stress, 12 strains of endophytic fungi with high antioxidant activity isolated from riparian plants *Myricaria laxiflora* were evaluated for their effects in rice by the crude extracts.

**Results:**

Of the 12 endophytic fungi, *Aspergillus fumigatus* SG-17 functioned most effectively, with the crude extract exhibiting relatively higher antioxidant capacity both in vivo and in vitro. The subsequent MS and NMR analysis showed that the primary substance responsible for the antioxidant activity in the extract was (Z)-N-(4-hydroxystyryl) formamide (NFA), an analogue of coumarin. Enzyme activity assay in nerve cells SH-SY5Y showed that NFA could maintain the membrane integrity and regulate the antioxidase activity under oxidative stress. In rice suffering drought stress, NFA effectively alleviated the harm by regulating the contents of NADPH oxidases, antioxidants and heat shock proteins, all of which are closely related with the reactive oxygen species pathway.

**Conclusion:**

These findings indicated that some endophytes from plants often subjected to flooding and oxidative stress could enhance drought resistance by producing compounds such as NFA to regulate the oxidative pathway.

**Electronic supplementary material:**

The online version of this article (10.1186/s12866-019-1419-5) contains supplementary material, which is available to authorized users.

## Background

Endophytic fungi can be beneficial to the host plant through evolutionary adaptation [13, 26]. They have a rich biodiversity [9, 32], and actively regulate the growth and development of the host or other plants [12, 40]. The metabolites present in endophytic fungi exhibit extensive physiological activity, such as anti-bacterial, anti-tumor, pesticidal, immunosuppressive and antioxidative effects [18, 32, 42]. Over the past few decades, endophytic fungi have become a potential resource for development of new drugs [10, 23]. In particular, they can improve the ability of plants to resist biotic or abiotic stresses [31, 38].

Drought is a yield limiting factor to rice [11, 15]. Physiological indexes such as Proline (Pro) content, malondialdehyde (MDA) content, membrane relative permeability (MRP), relative water content (RWC) and antioxidant enzyme activity are closely involved in drought response [45]. Under drought stress, the Pro and MDA content and MRP increase, while RWC declines. There is also a strong link between the damage by drought and the metabolic balance of reactive oxygen species (ROS) [21], such as hydrogen peroxide (H_2_O_2_), superoxide anion (O_2_•^−^), singlet oxygen (^1^O_2_) and hydroxyl radical (•OH) [21]. Therefore, to some extent, drought resistance is dependent on the antioxidative ability [21]. However, ROS has a dual function in plants [24]. On one hand, they can trigger oxidative bursts that improve resistance to stress [5]; on the other hand, they can induce membrane lipid peroxidation and cause a detrimental effect [33]. Thus, the dynamic equilibrium of ROS is crucial to plants, and the stronger the antioxidant capacity is, the higher the stress tolerance would be [33]. ROS is mainly produced by NADPH oxidase [39], and eliminated by both non-enzymatic antioxidants, such as polysaccharides, flavonoids, polyphenols, alkaloids, saponins and vitamins [18, 21, 41], and antioxidant enzymes, such as superoxide dismutase (SOD), catalase (CAT) and peroxidase (POD; [1, 33]).

Heat shock proteins (HSPs) are a class of proteins which are activated when plants are subjected to adversity [22]. As “molecular chaperones”, HSPs are often involved in folding, refolding, processing and transporting proteins [22]. Under drought stress, HSPs are actively involved in ROS metabolism in two ways: they affect the synthesis of ROS by inhibiting the activity of NADPH oxidase [14], and promote the expression of antioxidant enzymes related to ROS clearance and subsequent apoptotic metabolism [6, 14]. Thus, they have important applied value, and can provide a theoretical basis for selecting strongly resistant plant varieties [19]. Among them, HSP70 plays an important role in plant drought resistance [4, 29].

Recent reports have shown that the metabolites present in endophytic fungi are closely related to the habitat of the host plant [10]. *Myricaria laxiflora* is a shrub distributed specifically in the water draw-down zone around the Three Gorges Reservoir [34]. The plants can survive in nearly 6 months of seasonal flooding, indicating a strong adaptive ability to cope with deficient oxygen and the resulting oxidative stress [34]. As expected, in a previous study many endophytic fungi with high antioxidant activity were isolated from *M. laxiflora* [43].

*Aspergillus fumigatus* is commonly found in moist environments, negatively impacting yields and quality of crops [20]. Here, we found that the crude extract of endophytic *A. fumigatus* strain SG-17 had strong antioxidant activity, reaching 31.86% of that of vitamin C (Vc), and could help rice resist drought stress [8]. The main substance involved was an analogue of coumarin, named (Z)-N-(4-hydroxystyryl) formamide (NFA), which had been isolated from *Streptomyces amakusaensis* as a selective antibiotic treatment of *Mycobacterium* involved in tuberculosis [3]. NFA was also found in *A. finnigatus* as an inhibitor against rabbit platelet aggregation induced by arachidonic acid and collagen [35]. In the salt-tolerant fungus *Penicillium chrysogenum* from the Yellow River Delta [30] and in the marine-derived endophytic fungi [2], NFA was discovered to exhibit antibacterial activity [30], as well as moderate cytotoxicity against Du145, A-549 and Hela cell lines [2]. So far, there are few reports on the antioxidant activity of NFA. Here, we found that NFA from *A. fumigatus* was able to effectively alleviate drought stress in rice. This role may be mediated by regulation of oxidative pathway, involving antioxidant enzymes, HSP70 and NADPH oxidase. These findings indicate that the endophytic fungi from plants adapted to oxidative stress have a positive effect on plant resistance to drought stress.

## Methods

### Strain resource and medium

Twelve endophytic fungi from various tissues of *M. laxiflora* pre- and post- flooding [34] were used in a preliminarily screening (Table 1). With the permission of the local forestry administration, the plant samples were collected from the island of Yanzhiba, located in 109°32′E to 110°52′E and 30° 53′N to 31 °3′N. Dr. Wang YB of Biotechnology Center in China Three Gorges University carried out the formal identification, and the specimens (LSP201404220) was deposited in the Herbarium of Three Gorges University. All endophytic strains were stored at 4 °C in paraffin. The storage and activation medium was potato dextrose agar (PDA), comprising 200 g potato, 20 g glucose and 15–20 g agar, made up with distilled water to 1000 mL. The pH was adjusted to 6.0 and the medium was sterilized at 121 °C for 20 min. Sabouraud’s dextrose (SD) medium was used for liquid fermentation, which consisted of 10 g peptone and 40 g of glucose, made up with distilled water to 1000 mL. D-MEM/F-12 complete medium, used to culture nerve cells SH-SY5Y for the in vivo antioxidant activity assay, was sterilized by filtration through 0.45 μm microporous membranes [16].

**Table 1:** Characteristics of twelve endophytes isolated from *M. laxiflora*

Strain number	Tissue origin	Isolated condition^a^	T-AOC (U/mL) ^b^	Serial number in Genbank	The most homologous species^c^	Similarity (%)	Survival rate (%)^d^
HG-2	Root	AF	24.85 ± 0.22	MK450292	*Fusarium concentricum*	99	0
HY-1	Leaf	AF	34.88 ± 0.80	MK450293	*Aspergillus oryzae*	98	0
MG-23	Root	AF	21.32 ± 0.10	MK450294	*Aspergillus tubingensis*	99	0
MY-15	Leaf	AF	27.47 ± 0.92	MK450295	*Aspergillus flavus*	98	0
MY-22	Leaf	AF	18.56 ± 0.28	MK450296	*Alternaria alternata*	98	0
QY-1	Leaf	BF	55.90 ± 0.84	KU954091	*Chaetomium globosum*	100	23.33
SG-16	Root	BF	19.65 ± 0.13	MK450297	*Fomitopsis palustris*	96	0
SG-17	Root	BF	29.64 ± 0.17	MK450298	*Aspergillus fumigatus*	99	36.67
SG-4	Root	BF	15.85 ± 0.36	MK450299	*Penicillium oxalicum*	98	16.67
SG-5	Root	BF	20.20 ± 0.46	MK450300	*Alternaria tenuissima*	99	0
SG-6	Root	BF	21.03 ± 0.17	MK450301	*Pestalotiopsis microspora*	99	0
SY-15	Leaf	BF	25.99 ± 0.47	KX822145	*Penicillium oxalicum*	98	13.33
Average T-AOC of 163 fungi			8.22 ± 1.76	–	–	–	–
NFA			–	–	–	–	50
Proline (Pro)			–	–	–	–	30
Ascorbic acid (Vc)			–	–	–	–	0

^a^Note: AF means that the fungus was isolated from *M. laxiflora* plants after flooding, and BF before flooding. ^b^T-AOC: Total antioxidant capacity. ^c^The most homologous species are obtained by ITS sequence alignment in Genbank [34]. ^d^Survival rate was investigated by applying the crude extract of endophytic fungi or other substances to 30 rice seedlings after drought

### Preparation of the crude extract

Hyphae of the 12 endophytic fungi were inoculated individually in 150 mL of SDA liquid medium, and then grown for 14 d at 28 °C. Fungi in the fermented liquid were harvested by suction and extracted 3 times with an equal volume of ethyl acetate. The organic phases were combined and distilled under vacuum at 40 °C to harvest the extraction of ethyl acetate.

### Antioxidant activity measurement

Both in vitro and in vivo methods were employed to evaluate the antioxidative ability. For in vitro assessment, total antioxidant capacity (T-AOC) kit [28, 43] and 1, 1-diphenyl-2-trinitrophenylhydrazine (DPPH) radical scavenging kit [36] were used to screen the fermented broth and the crude extract, respectively, by following the manufacturer’s instructions. Both kits were from Nanjing Jiancheng Bioengineering Institute, Nanjing, China (http://www.njjcbio.com/). The in vivo antioxidant activity was determined by nerve cells SH-SY5Y according to the manufacturer’s instructions (Beyotime Biotechnology Company, Shanghai, China, http://beyotime.bioon.com.cn/) [16]. In order to characterize the possible mechanism of antioxidant protective effect on nerve cells, we determined cysteine-dependent aspartate-directed protease (Caspase) 3, Caspase 9, SOD and lactate dehydrogenase (LDH) activity [16]. LDH leakage rate was regarded as a reliable indicator of cellular membrane integrity, and calculated as [16]: OD value of the supernatant in the medium/OD value of the total cells × 100%, and data were normalized from control (100%). All the results were replicated 3 times.

### Drought resistance analysis in rice

Seeds of rice cultivar Nipponbare were placed first in 1% nitric acid for 12 h and then in tap water for 2 d to break dormancy. The rooting seeds were planted into the plastic cups or pots containing about 100 g of local soil, and then grew in a light incubator (30 °C, 30,000 lx light intensity and approximately 60% relative humidity) for 20 d. The volume of the cup was 245 cm^3^ with a height of 9.5 cm. Before transferred to the cups, the soil had been air-dried and well-mixed. After 20 d, rice seedlings with similar size were selected and grown for another 20 d under drought stress. One day before drought treatment, 50 mL of 0.1 mg/mL treatment solution (crude extract or NFA) or the living hyphae were applied to the seedlings. Proline solution at 50 μM was used as a positive control. In order to compare the effects of other antioxidants, 0.1 mg/mL Vc solution was applied to rice seedlings. In the drought period, seedlings were deprived of water. Each treatment was replicated 3 times.

### Physiological indexes investigation

After 20-d drought stress at room temperature, the survival rate of rice seedlings and physiological indexes were investigated. Pro and MDA contents were assayed using kits from Nanjing Jiancheng Bioengineering Institute (Nanjing, China). Analysis of SOD, POD, HSP70 and NADPH oxidase was preformed using kits from Jiangsu Baolai Biotechnology Co., Ltd. (Jiangsu, China). RWC was calculated based on the equation: RWC (%) = [(Fresh weight − dry weight)/(turgid weight − dry weight)] × 100 [7]. MRP (%) was measured according to published methods [45], and calculated as: Conductivity of leakage before death/ Conductivity of leakage of after death× 100.

### Isolation and identification of the primary substance from SG-17

The SG-17 crude extract was firstly separated by preparative thin layer chromatography (Pr-TLC) with the expansion condition of petroleum ether: acetone = 1: 1 (*V*/V). Four bands were visible when the Pr-TLC plate was held under a UV 254 nm lamp. These bands were eluted by methanol to obtain four fractions (Fraction1~4, respectively). After in vitro antioxidant activity analysis using T-AOC and DPPH method, fraction 2 with the strongest activity was separated and purified by semi-preparative high performance liquid chromatography (HPLC) (250 × 10 mm id, Cosmosil MS-II). The conditions were as follows: mobile phase, acetonitrile: water = 60:40 (V/V) with a flow rate of 3.0 mL/min and a UV detection wavelength of 254 nm. The isolated and purified compounds by HPLC were freeze-dried, then dissolved in 0.5 mL deuterium DMSO. ^1^H-NMR and ^13^C-NMR were determined by Bruker AVANCE 400 MHz nuclear magnetic resonance (NMR) spectroscopy. All reagents were analytical grade, except that acetonitrile and methanol used for high HPLC were chromatography-grade. The water used was triple-distilled.

### Data processing

The data were expressed as mean ± standard deviation. Significant differences among groups were calculated using One-Way ANOVA, followed by multiple comparisons using Ducan’s test, provided by the statistical software SPSS 20.0 (For windows version, SPSS Inc., Chicago, IL, USA).

## Results

### Preliminary screening of fungi assisting rice against drought stress

**Fig. 1:**
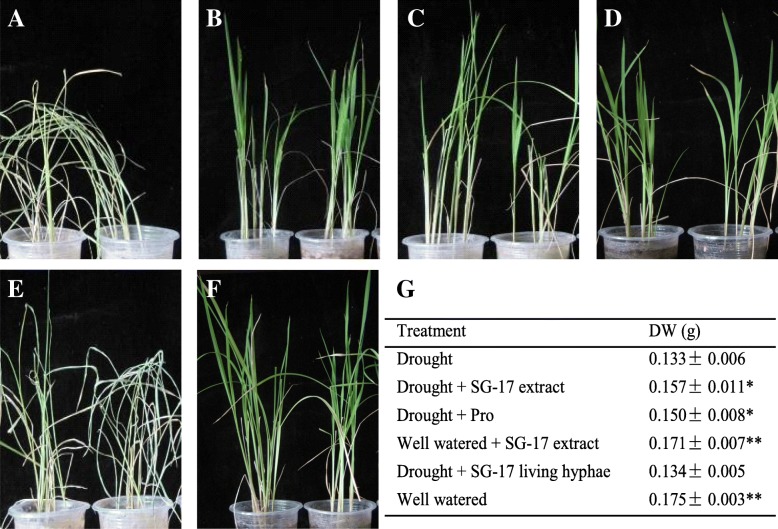
Effect of SG-17 crude extract on rice against drought stress. **a** Rice seedlings after 20 days of drought. **b** Drought + SG-17 crude extract. **c** Drought + Proline. **d** Well watered + SG-17 crude extract. **e** Drought + SG-17 living hyphae. **f** Well watered seedlings. **g** Dry weight (DW) of the seedlings, * means significant difference with *P* < 0.05 and ** for *P* < 0.01 compared with drought group, *n* = 3

In the previous study, 163 endophytic fungi from various tissues of *M. laxiflora* pre- and post- flooding were isolated [34]. Here, 12 strains were selected for their relatively high antioxidant activity (43, Table 1). To evaluate the contribution to drought tolerance in rice, the living hyphae or the crude extracts of 12 fungi were applied to the seedlings. After 20-d drought stress at room temperature, none of the living hyphae had any effect on drought response (Fig. 1). However, crude extracts of QY-1, SG-4, SG-17, and SY-15 increased the survival rate of rice under drought stress (Table 1). The most effective strain was SG17, identified as an *A. fumigatus* [9], by which the effect was nearly equivalent to the positive control of Proline (Fig. 1). Besides, its crude extract did not affect the normal growth of rice (Fig. 1).

### Effects of SG-17 crude extract on rice susceptibility to drought

**Fig. 2:**
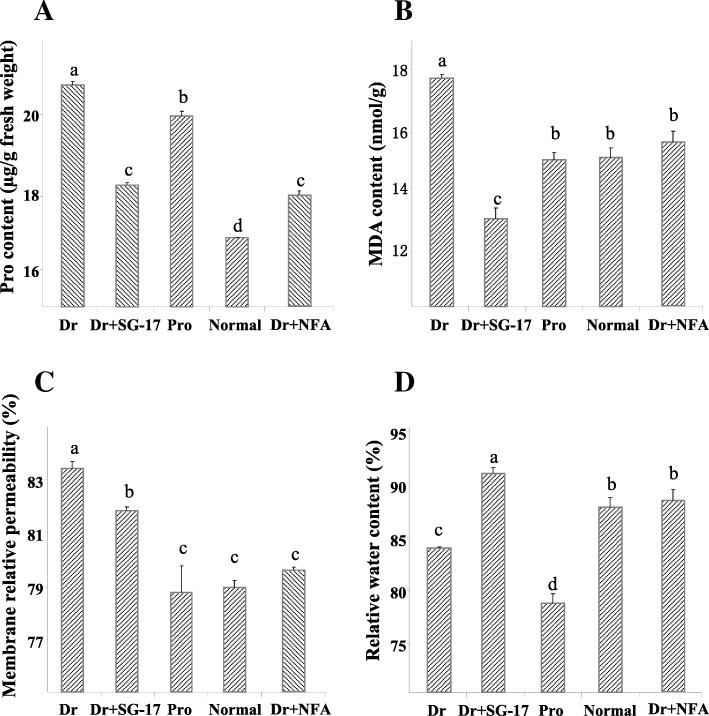
Physiological analysis of rice against drought by SG-17 crude extract. **a** Proline content. Dr.: seedlings subjected to drought for 20 days. Dr. + SG-17: Drought + SG-17 crude extract. Pro: Drought + Proline. Normal: well watered. Ducan’s multiple range test, *p* < 0.05 and *n* = 3. **b** MDA content. **c** Membrane relative permeability. **d** Relative water content

To analyze the effects of SG-17 crude extract, we investigated some physiological indexes of rice seedlings after 20 days drought treatment. Under drought conditions, Proline content of rice seedlings treated with the crude extract was significantly lower than that of the untreated control, and was even lower than that of seedlings treated with Proline (Fig. 2a). Similar results were found on the indexes of MDA and MRP (Fig. 2b and c), indicating that SG-17 crude extract could effectively enhance drought tolerance of rice. This effect was even better than the positive control of Proline. The conclusion was further supported by the result of RWC, which was significantly higher in the crude extract treated samples than in the untreated or positive (+Pro) control (Fig. 2d). It was also noted that RWC in the seedlings of Dr. + SG17 was even higher than in those well watered, implying that SG-17 extracts could trigger a certain water metabolism to keep rice away from drought.

### Antioxidant activity of SG-17 crude extract and the constituents in vitro

The antioxidant activity of SG-17 crude extract was measured by the T-AOC kit and the DPPH radical scavenging kit in vitro. The extract showed a certain degree of antioxidant activity. Although the T-AOC value of the extract was lower than Vc, the DPPH free radical scavenging rate reached 90.57%, almost equivalent to that of Vc (Table 2). After separation by thin layer chromatography and HPLC, four fractions were purified from the extract. Through tracing the antioxidant activity, we found that all four fractions exhibited a certain degree of antioxidant ability. Among them, fraction 2 had the highest activity, with the T-AOC value over 16 times more than that of Vc (Table 2).

**Table 2:** Antioxidant activity of SG-17 crude extract assayed by two methods

Substances	T-AOC(U/mL)^a^	DPPH Clearance rate(%)
SG-17 crude extract	62.56 ± 0.16^**^	90.57 ± 0.35^**^
Vc as a control	196.33 ± 0.11^**^	91.87 ± 0.05^**^
Fraction 1	0.00 ± 0.05	3.42 ± 0.16^**^
Fraction 2	3240.94 ± 25.99^**^	23.79 ± 0.27^**^
Fraction 3	0.00 ± 0.03	11.38 ± 0.25^**^
Fraction 4	769.67 ± 12.59^**^	0.00 ± 0.05
Blank	0.00 ± 0.07	0.00 ± 0.03

^a^ANOVA analysis was compared with the blank group at *n* = 3, and ^*^ means significant difference at *P* < 0.05, ^**^ for *P* < 0.01

### Antioxidant activity of fraction 2 in vivo

**Fig. 3:**
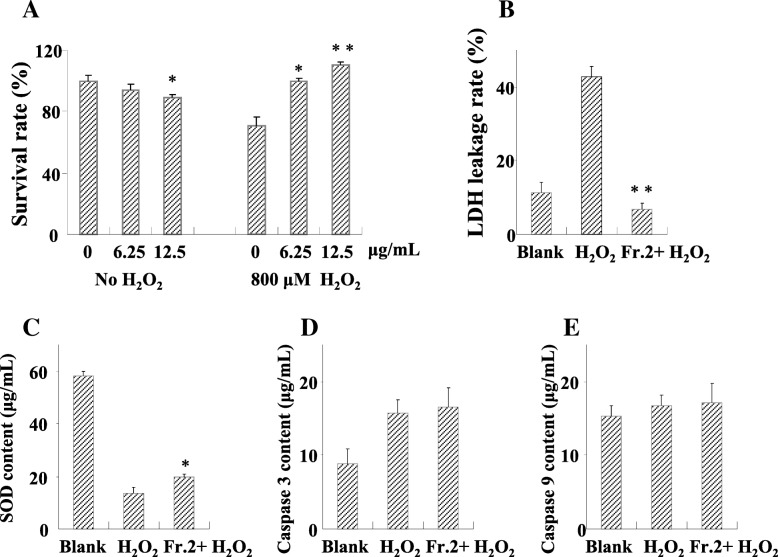
Effect of fraction 2 in protecting SH-SY5Y cells from oxidative stress. **a** Various concentration of fraction 2 and the resultant relative survival rate with or without simulated oxidant, t-test, * means *P* < 0.05, and ** for 0.01. **b** Leakage rate of lactate dehydrogenase under oxidant stress. Enzyme activity of SOD (**c**), Caspase 3 (**d**) and Caspase 9 (**e**) response to simulated oxidant and fraction 2

We determined the antioxidant protective effect of fraction 2 on nerve cells SH-SY5Y subjected to oxidative stress induced by 800 μM H_2_O_2_. Although, under normal conditions without H_2_O_2_, fraction 2 at 12.50 μg/mL showed some toxicity to cell growth (Fig. 3a), it increased the relative survival rate of SH-SY5Y by 56.49% in the presence of oxidative stress (Fig. 3a), exhibiting an antioxidant protective effect on nerve cells. This result suggested that the underlying substance in fraction 2 may function through alleviating the oxidative damage.

In order to identify the potential antioxidant mechanism, we measured the LDH leakage rate and enzyme activity of SOD, apoptotic protein caspase 3 and caspase 9 in SH-SY5Y cells upon treatment of 12.50 μg/mL fraction 2. We found that fraction 2 decreased the LDH leakage rate by 81.5% under oxidative stress (Fig. 3b), suggesting that fraction 2 could maintain membrane integrity (Fig. 3b). The activity of SOD was also enhanced by fraction 2 treatment under oxidative stress (Fig. 3c), while that of caspase 3 or caspase 9 was not affected (Fig. 3d and e). These results suggested that fraction 2 may be involved in antioxidant metabolic pathways mainly through maintaining membrane integrity and regulating the activity of antioxidant enzymes.

### Structure determination of NFA

**Fig. 4:**
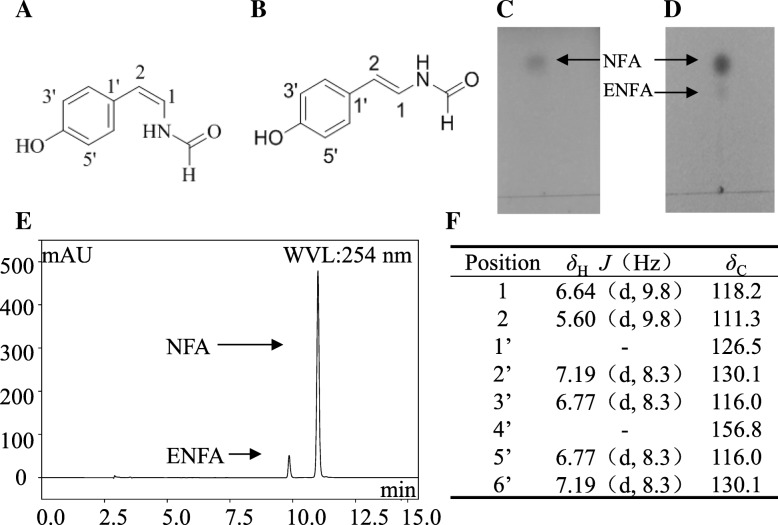
Identification of fraction 2 as NFA. **a** Structure of NFA. **b** Structure of ENFA. Separation of NFA (**c**) and ENFA (**d**) by TLC. **e** Separation of fraction 2 by semi-preparative HPLC. **f** ^1^H-NMR and ^13^C-NMR data of compound fraction 2

Based on the above results, we speculated that fraction 2 isolated from SG-17 crude extract by thin layer chromatography (TLC) might contain the main constituent responsible for the antioxidant activity. Chromatographic analysis showed that the purity of this fraction had got to 98%. After MS and NMR analysis, the structure of fraction 2 was confirmed as a coumarin analogue named (Z)-N-(4-hydroxystyryl) formamide (NFA, Fig. 4a) according to the spectral characteristics (Fig. 4f), which was consistent with previous data [30, 35]. Moreover, it was reported that NFA can gradually transform to an isomer (E)-N-(4-hydroxystyryl) formamide (ENFA) ([30, 35], Fig. 4b). Here, similar results were found. In TLC seperation, ENFA appeared below NFA after 1 day at room temperature (Fig. 4c and d). In semi-preparative HPLC analysis, both compounds were eluted almost together (Fig. 4e), with the retention time of ENFA being about 9.58–10.00 min and that of NFA about 10.78–11.30 min (Fig. 4e).

### Effect of NFA on rice resistance to drought stress

**Fig. 5:**
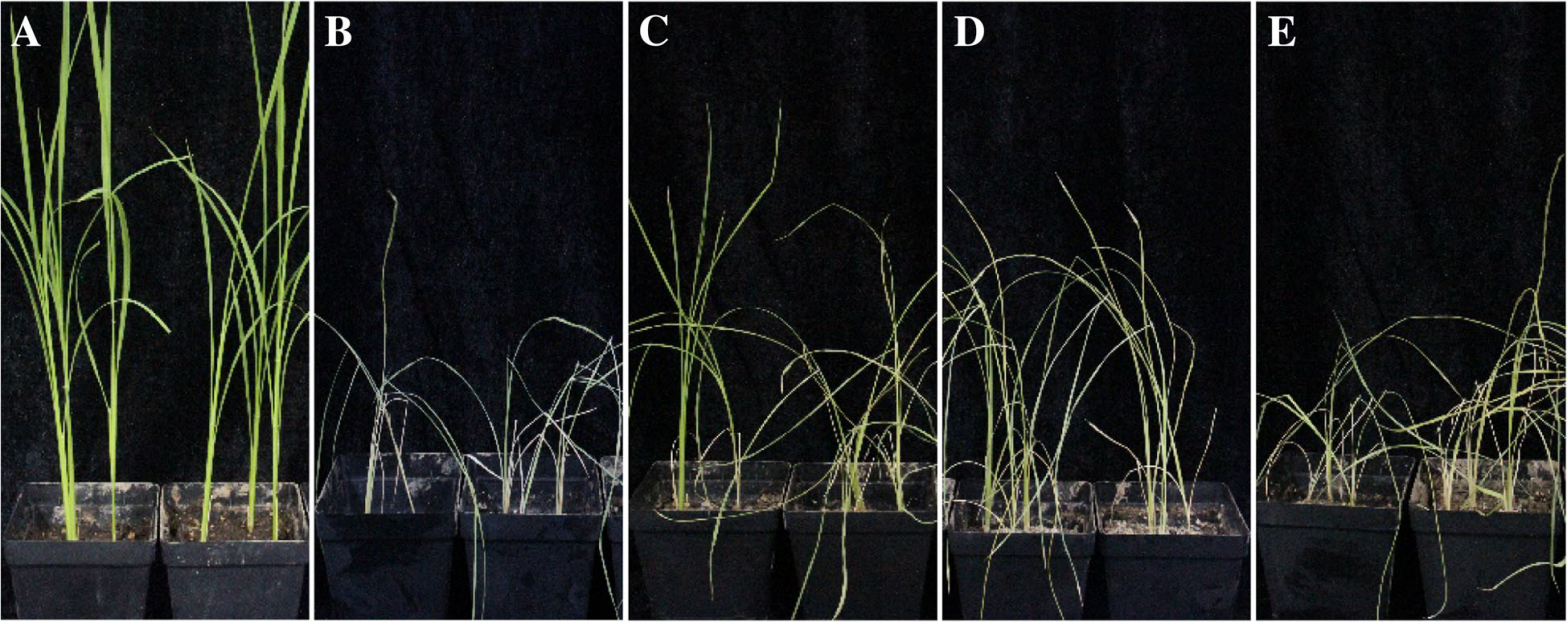
Effects of NFA on rice resistance to drought stress. **a** Well watered rice seedlings. **b** Seedlings under drought. **c** Drought + NFA. **d** Drought + Proline. **e** Drought + Vc

In order to prove that NFA is responsible for the effects against drought stress, we applied the purified NFA solution directly to rice seedlings. After drought for 20 days, most seedlings even treated by Vc died, whereas some in the NFA treatment survived and grew better than that of the positive control Proline (Fig. 5), indicating that NFA could effectively protect rice from drought damage. To an extent, the effect of NFA was superior to that of the osmotic modulator, Proline. After applying NFA, the contents of Pro and MDA were significantly lower than those of other treatments under drought stress (Fig. 2a and b). Besides, NFA decreased the membrane relative permeability (Fig. 2c), indicating a protective effect on membrane integrity under drought. Moreover, during drought stress, NFA maintained relatively high water content in rice leaves (Fig. 2d). These results proved that NFA was able to effectively assist rice in drought resistance.

### NFA regulated oxidative pathway of rice under drought stress

**Fig. 6:**
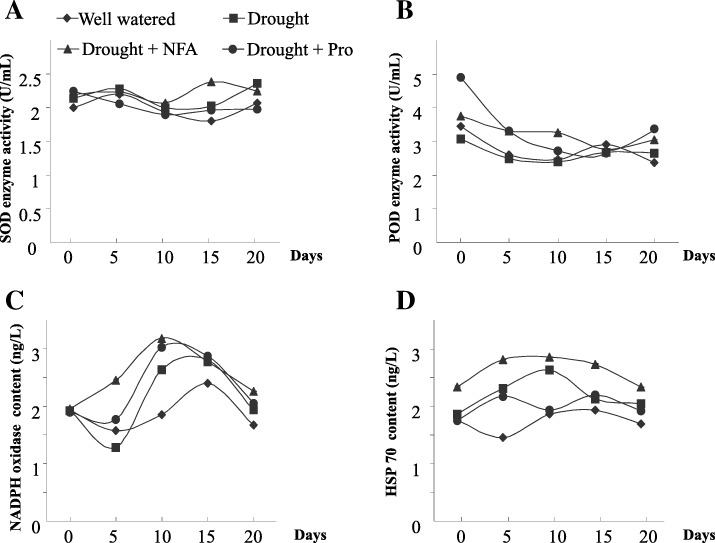
Oxidative metabolism affected by NFA in rice under drought adversity. Dynamic data were acquired after drought for 5, 10, 15 and 20 days. **a** SOD enzyme activity. **b** POD enzyme activity. **c** NADPH oxidase content. **d** HSP70 content

It has been known that drought is closely associated with oxidative stress [15, 39]. To interrogate the relationship between NFA and the oxidative pathway under drought adversity, we measured the dynamic activity of SOD, POD, HSP70 and NADPH oxidase in rice seedlings stressed by drought. After 15 days of drought, the enzyme activity of SOD was significantly higher for NFA-treated group (*P* < 0.01) compared with other groups (Fig. 6a). The variation of POD enzyme activity had a similar tendency after 10 days (Fig. 6b). Both SOD and POD are important scavengers of ROS induced by drought [33], therefore, NFA could help rice resist drought possibly by regulating antioxidant enzymes involved in the oxidative pathway.

Once plants suffer from drought, NADPH oxidase will be induced to produce a certain range of ROS [39]. Here, the NADPH oxidase content after treatment with NFA was significantly increased (*P* < 0.01), especially after 5 days of drought (Fig. 6c). This result indicated that in early drought, NFA could upregulate NADPH oxidase to produce ROS and subsequently participate in the anti-stress metabolism. In early drought not longer than 5 days, NFA also significantly induced the expression (*P* < 0.01) of HSP70 (Fig. 6d), which is known to be extensively involved in the metabolism of ROS [4, 19]. This result further suggested that NFA enhanced drought resistance in rice through regulation of oxidative pathway.

## Discussion

Recent studies have shown that endophytic fungi in plants are species-rich, and show great utilization potential in medicine, food and agriculture [37, 44]. In this study, we showed that the endophytic fungus *A. fumigatus* isolated from *M. laxiflora*, a plant tolerant to hypoxia stress, exhibited strong in vivo and in vitro antioxidant activity. By producing the active compound NFA, the fungus could effectively assist rice to resist drought.

To our knowledge, by far no one has reported the antioxidant activity of NFA, nor its effect on drought resistance in plants. In this study, we identified an antioxidant NFA from the endophytic fungus *A. fumigatus* and found that it could maintain membrane integrity, and regulate the contents of NADPH oxidase, antioxidases and HSPs. As NADPH oxidase directly controlls the ROS production, whereas antioxidase and HSPs affect the degradations of ROS, NFA alleviating drought stress in rice may be mediated by regulation of oxidative pathway. Our physiological data indicated that the drought resistance of rice conferred by NFA might be attributed to NFA’s dual effects: it induced NADPH oxidase after a short period of drought, while activated antioxidant enzyme system to eliminate ROS after a long period of drought (Fig. 6c and d). These findings are consistent with the dual role of ROS, implying that NFA may regulate the homeostasis of ROS in a drought stage-dependent manner.

In addition to *A. fumigatus* SG-17, other endophytic fungi, such as SG-4, SY-15 and QY-1, could help rice resist drought stress as well. In rice anti-flooding test, we obtained similar results (data not shown). Thus, endophytic fungi, especially those in the special habitat plant *M. laxiflora*, are of potential application value in oxidative stress. It is worth to mention that although SG-17 did not show the highest antioxidant activity, it generated the best effect of enhancing the drought resistance of rice among the 12 endophytic fungi tested.

In identifying the structure of fraction 2, the antioxidant activity determined by two in vitro methods of T-AOC and DPPH differed. Although NFA exhibited relatively high T-AOC value, its free radical scavenging rate was lower. The possible reason was that when measured by DPPH method, some substances might be generated in the process, which deepened the solution color (Additional file 1: Figure S1), resulting in less photometric count.

In this study, both HSP70 and NADPH oxidase in rice were induced by NFA at the early stages of drought stress. Hence, HSP70 is unlikely to inhibit the synthesis of NADPH oxidase. The roles of HSP70 in the antioxidative pathway need further research. Meanwhile, there are a series of enzymes associated with oxidative stress, such as mitogen-activated protein kinase (MAPK) [25], rho-related GTPase from plants (ROP) [27], apoptosis proteins [17], and so on. To further clarify the mechanism of drought resistance mediated by NFA, activities of these downstream enzyme families need to be studied in the future.

## Conclusions

To maintain stable crop yields and guarantee global food security, it is of great significance to improve drought resistance via clarifying the underlying mechanisms. Here, a new antioxidant, NFA, could alleviate drought stress in rice by regulation of oxidative pathway. The antioxidant activity and the physiological effects on plants of NFA were analyzed in detail, potentially providing a new clue for antioxidant development by chemiecology and for our understanding of the symbiosis between endophytic fungi and host plants subjected to oxidative stress.

## Additional file

Additional file 1: **Figure S1.** Antioxidant activity of fraction 2 by DPPH method. (A) DPPH alcohol solution. (B) fraction 2 solution. (C) Vc solution. (PPT 6256 kb)

## Abbreviations

*A. fumigatus*: *Aspergillus fumigatus*
Caspase: Cysteinyl aspartate specific proteinase
DPPH: 1, 1-diphenyl-2-picrylhydrazyl
ENFA: (E)-N-(4-hydroxystyryl) formamide
HPLC: High performance liquid chromatography
HSP: Heat shock proteins
LDH: Lactate dehydrogenase
*M. laxiflora*: *Myricaria laxiflora*
MDA: Malondialdehyde
MRP: Membrane relative permeability
NFA: (Z)-N-(4-hydroxystyryl) formamide
PDA: Potato dextrose agar
POD: Peroxidase
Pro: Proline
ROS: Reactive oxygen species
RWC: Relative water content
SD: Sabouraud’s dextrose
SOD: Superoxide dismutase
T-AOC: Total antioxidant capacity
Vc: Vitamin C
