# Supplementary material for: A coumarin analogue NFA from endophytic Aspergillus fumigatus improves drought resistance in rice as an antioxidant
Source: BMC Microbiol. 2019 Feb 26;19:50. doi: 10.1186/s12866-019-1419-5 (PMC6390358; doi:10.1186/s12866-019-1419-5)
Supplement: Supplementary file 1 — Figure S1. Antioxidant activity of fraction 2 by DPPH method. (A) DPPH alcohol solution. (B) fraction 2 solution. (C) Vc solution. (PPT 6256 kb) [file 12866_2019_1419_MOESM1_ESM.ppt]

## Slide 1
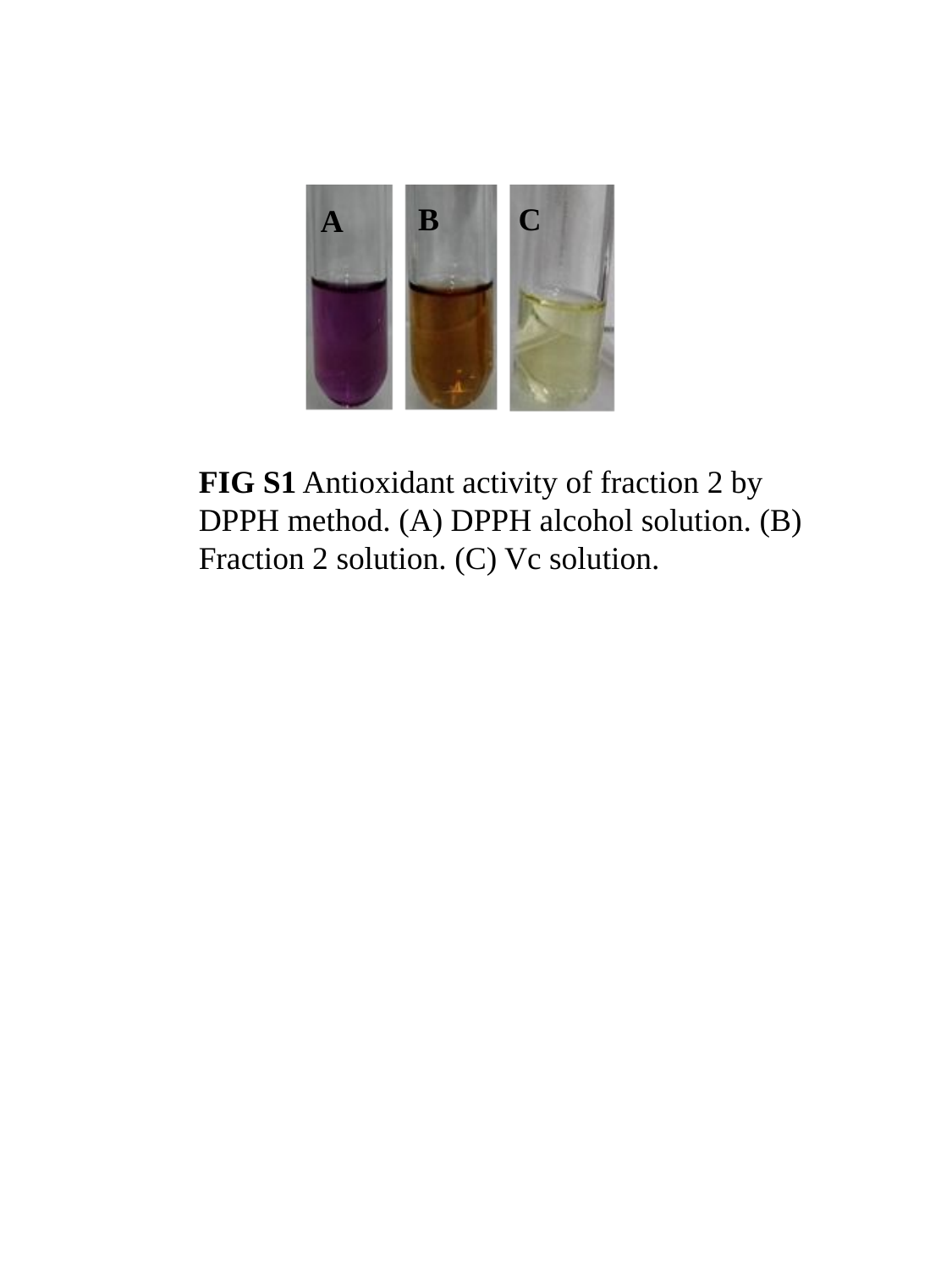

B
C
A
FIG S1 Antioxidant activity of fraction 2 by DPPH method. (A) DPPH alcohol solution. (B) Fraction 2 solution. (C) Vc solution.
